# Perioperative severe airway hemorrhage during veno-arterial extracorporeal membrane oxygenation for acute massive pulmonary embolism: A case report

**DOI:** 10.1097/MD.0000000000050401

**Published:** 2026-08-28

**Authors:** Kun Cao, Feng Hai

**Affiliations:** a Department of Critical Care Medicine, Dalian City Third People’s Hospital, Dalian, China.

**Keywords:** airway hemorrhage, case report, extracorporeal membrane oxygenation (ECMO), massive pulmonary embolism (MPE)

## Abstract

**Rationale:**

According to the 2019 ERS/ESC guidelines, high-risk pulmonary embolism is defined as a case that presents with hemodynamic instability, obstructive shock, or cardiac arrest, and this condition has an extremely high death rate. ECMO can provide temporary circulatory support, acting as a bridge to definitive reperfusion therapy.

**Patient Concerns:**

The patient is a 25-year-old woman who had previously used estrogen-containing oral contraceptives. She sustained trauma to her right lower limb, necessitating prolonged immobilization. One month before her symptoms started, she had a high fever. One hour before she was admitted to the hospital, she fainted and went into cardiac arrest. Ten minutes of cardiopulmonary resuscitation were needed before her spontaneous circulation returned.

**Diagnoses:**

The electrocardiography test showed an S_1_Q_3_T_3_ pattern. An ultrasonography check confirmed thrombosis in the right popliteal vein. Transthoracic echocardiography found that the right heart was enlarged, and the left ventricle had a D shape. Computed tomography pulmonary angiography showed a saddle embolus right at the bifurcation of the pulmonary artery, which confirmed acute massive pulmonary embolism (MPE).

**Interventions:**

The patient was supported with veno-arterial extracorporeal membrane oxygenation (VA-ECMO), followed by catheter-directed thrombolysis and targeted airway hemostasis. Urokinase was administered through 2 thrombolysis catheters, and anticoagulation was adjusted dynamically according to bleeding and coagulation status.

**Outcomes:**

After the procedure, airway bleeding was stopped using targeted hemostasis methods, and the patient was able to come off ECMO successfully after 73 hours of use.

**Lessons:**

In this case, we achieved successful support from VA-ECMO for a patient with acute MPE. Our work shows that even severe bleeding in the airway can be handled safely, and you don’t have to stop anticoagulation treatment to do it. This approach ended up with the patient making a full recovery in the end.

## 1. Introduction

The annual incidence of pulmonary embolism is approximately 39 to 115 per 100,000 individuals,^[1]^ and the global annual incidence of venous thromboembolism exceeds 10 million cases.^[2]^ The 2019 European Respiratory Society/European Society of Cardiology guidelines define any cases of pulmonary embolism accompanied by hemodynamic instability, obstructive shock, persistent hypotension, or cardiac arrest as high-risk pulmonary embolisms, which are associated with extremely high mortality.^[3]^

In patients with massive pulmonary embolism (MPE) requiring ECMO who develop airway hemorrhage, the conventional approach of discontinuing anticoagulation carries substantial thrombotic risk. Here, we report a case in which continued anticoagulation and thrombolysis, combined with targeted vasopressin-mediated hemostasis, was successfully employed. To our knowledge, it has not been previously described.

## 2. Case presentation

### 2.1. Ethical approval and informed consent

This case report was reviewed and approved by the Ethics Committee of Dalian City Third People’s Hospital (Approval No. 2026-039-001). As this study followed a retrospective case analysis design and patient data were anonymized, the requirement for informed consent was waived.

### 2.2. Case history

**Figure 1.**
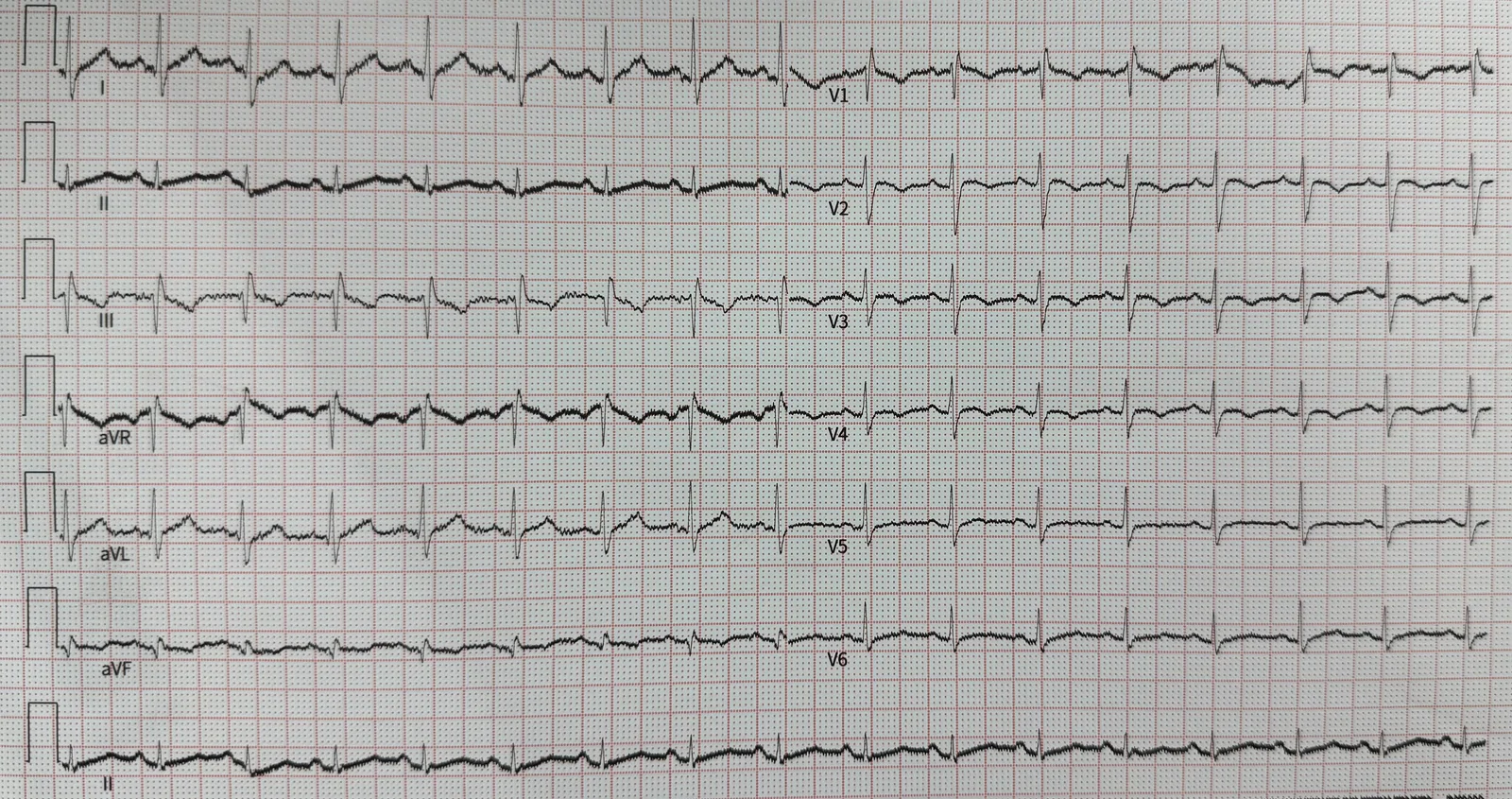
Electrocardiogram results on admission demonstrating an S_1_Q_3_T_3_ pattern.

**Figure 2.**
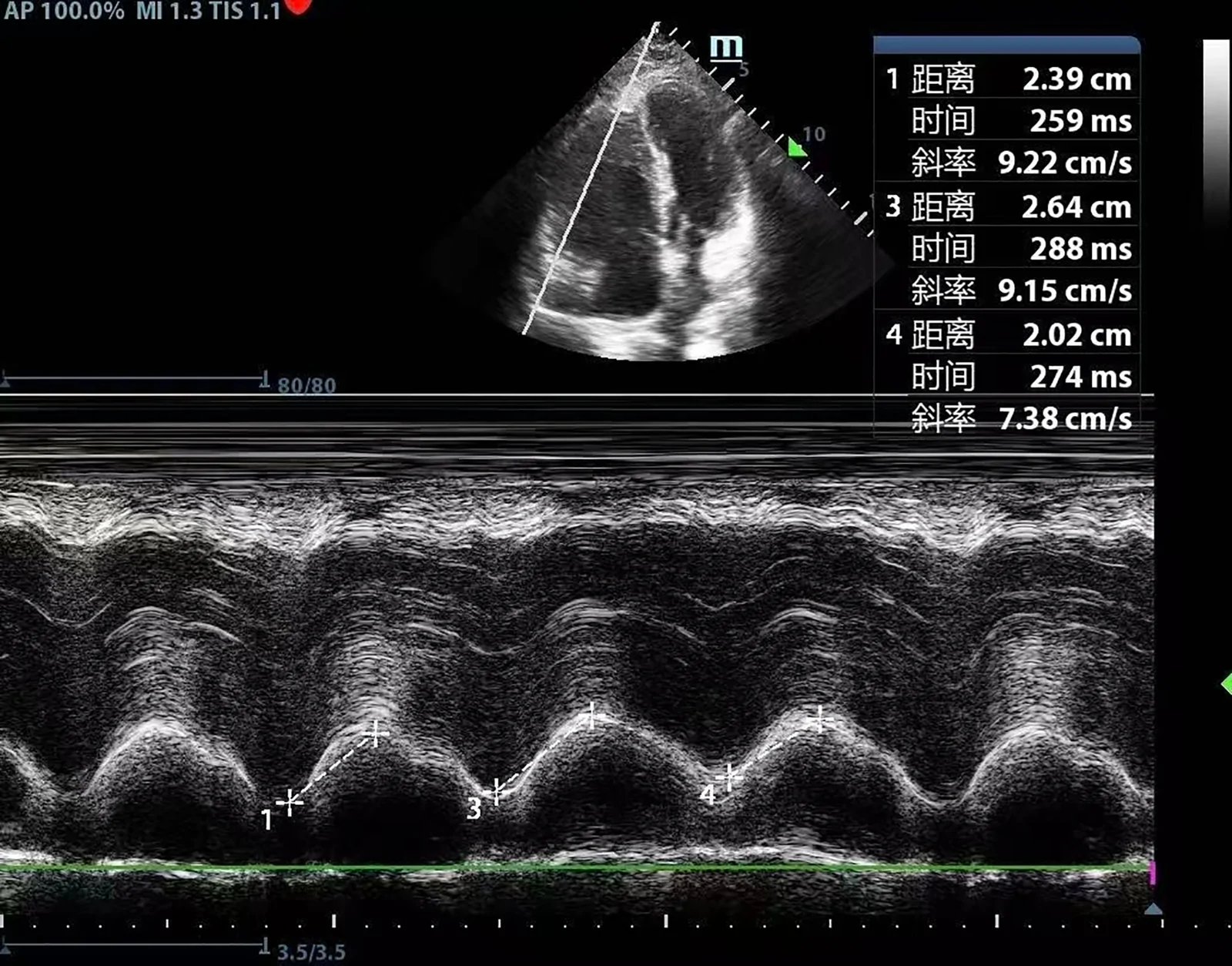
Transthoracic echocardiography on admission showing right heart enlargement, a D-shaped left ventricle, and impaired right ventricular function.

A 25-year-old woman with a history of estrogen-containing oral contraceptive use presented with multiple risk factors, including right lower limb trauma, prolonged sedentary behavior, and high fever within one month prior to onset. One hour prior to admission, she suddenly lost consciousness. Cardiopulmonary resuscitation was given for 10 minutes at the accident scene, which brought back spontaneous circulation. After that, she was taken to the emergency department immediately. The physical exam showed that her Glasgow Coma Scale score was 3. Both of her pupils were equal, round, and around 3.0 mm across. Her heart rate was 140 beats per minute, and mean arterial pressure (MAP) was only 45 mm Hg. Because of the very low pressure, she had to get norepinephrine at 1.45 μg·kg^−1^·min^−1^. She was on mechanical ventilation with 100% oxygen, but her pulse oxygen saturation was still just 60%. Lab tests showed a troponin I level of 0.02 ng/mL and a D-dimer level of 21.22 mg/L. A test for serum influenza A virus-specific IgM antibody was positive. Electrocardiography showed a clear S_1_Q_3_T_3_ pattern, as shown in Figure 1. An ultrasound scan found that a thrombosis had formed in her right popliteal vein. Transthoracic echocardiography showed that her right ventricular end-diastolic anteroposterior diameter (taken from the parasternal long-axis view) was 27 mm, and the right ventricular basal end-diastolic diameter (from the apical four-chamber view [A4C]) was 52 mm; this is shown in Figure 2.

**Figure 3.**
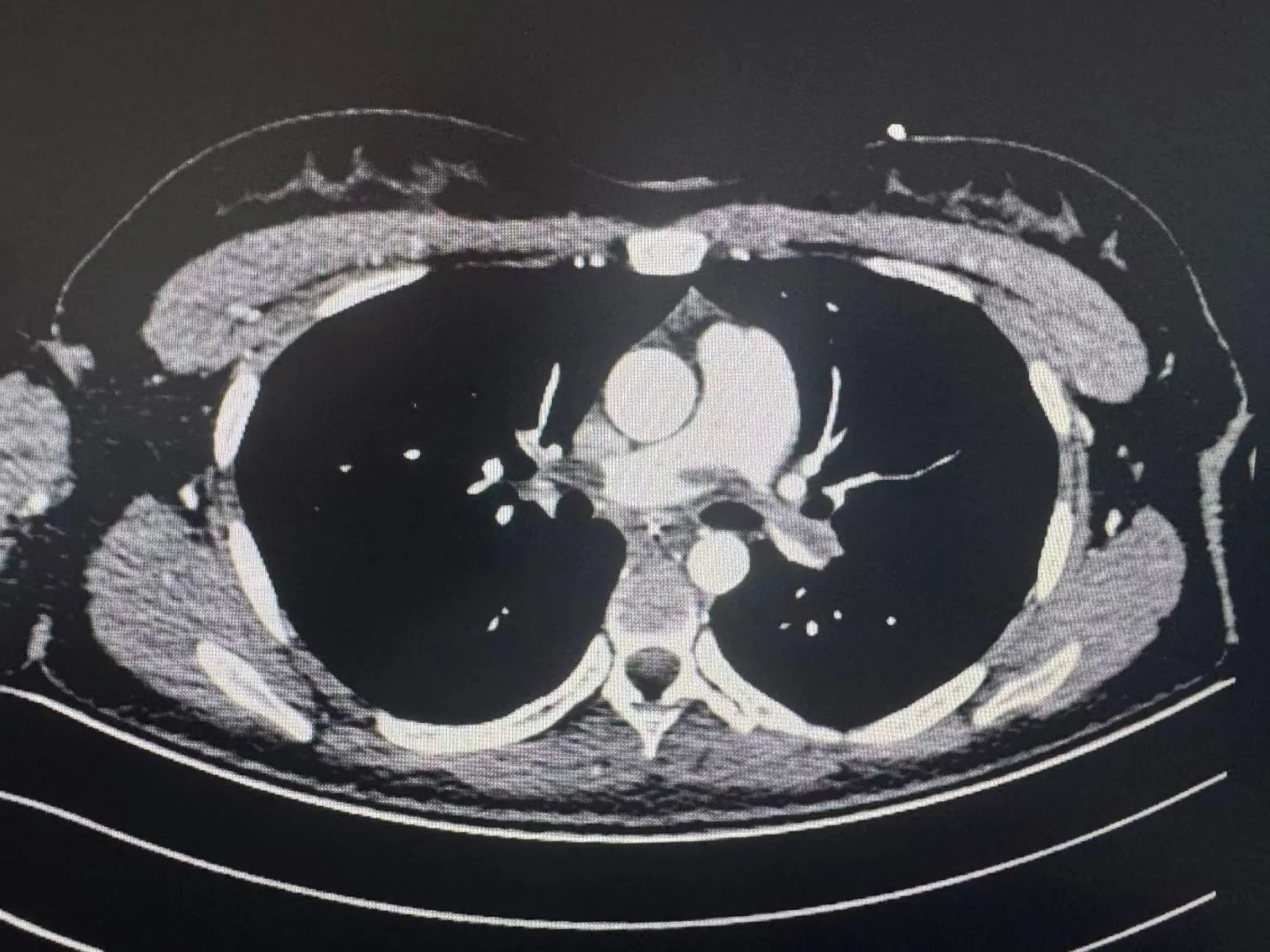
CTPA after admission demonstrating a saddle embolus at the bifurcation of the main pulmonary artery. CTPA = computed tomography pulmonary angiography.

The parasternal short-axis view showed that the patient’s left ventricle had a D-shaped structure. The speed of tricuspid regurgitation was more than 4 m/s, and the pressure gradient was 69 mm Hg. The left ventricular ejection fraction was measured at 45%. After the patient was transferred to the intensive care unit, MAP was 50 mm Hg with norepinephrine at 1.11 μg·kg^−1^·min^−1^, and the vasoactive-inotropic score (VIS) was 111. For mechanical ventilation, we set the fraction of inspired oxygen at 100% and added a positive end-expiratory pressure of 7 cm H_2_O. About an hour after she was admitted to the hospital, we started treatment with veno-arterial extracorporeal membrane oxygenation (VA-ECMO). The right femoral artery and vein were placed in an anteroposterior position. We performed cannulation through the left femoral vein and artery, using BE-PVL21/55 and BE-PVL17/23 cannulas, respectively. We placed a 6F Terumo sheath into the left superficial femoral artery, then connected it through a three-way stopcock to the side port of the ECMO arterial line to provide distal limb perfusion. The initial ECMO settings were as follows: pump speed was set to 4500 rpm; blood flow was between 4.33 and 4.52 L/min; the fraction of oxygen given was 100%; and sweep gas flow was at 4.5 L/min. One hour after we started ECMO, MAP went up to 65 mm Hg, and norepinephrine reduced to 0.55 μg·kg^−1^·min^−1^, while VIS decreased to 55. Pump speed was subsequently reduced to 4000 rpm, with a flow of 4.0 L/min. After 44 hours of VA-ECMO support, computed tomography pulmonary angiography showed a strip-like filling defect in the main pulmonary artery straddling the bifurcation of the left and right pulmonary arteries. The diameter of the main pulmonary artery was 26 mm, while the ascending aorta measured 23 mm at the same level. These results confirmed the diagnosis of acute MPE (Fig. 3).

After 47.5 hours of ECMO support, pulmonary angiography was performed, followed by balloon dilation and catheter-directed thrombolysis. Two thrombolysis catheters were placed in the right upper pulmonary artery and left lower pulmonary artery. A total of 200,000 U urokinase was administered into each pulmonary artery through the 2 catheters at a rate of 10,000 U/min, followed by continuous infusion of 8000 U/h through each catheter. Two hours following the interventional procedure, the patient developed airway hemorrhage characterized by bright red blood, with an estimated volume of 15 to 30 mL/h, consistent with continuous mild-to-moderate bleeding. MAP decreased from 71 to 51 mm Hg. Local hemostasis was achieved with topical thrombin, and red blood cell transfusion was administered. Vasoactive support included norepinephrine at 0.53 μg·kg^−1^·min^−1^ and epinephrine at 0.13 μg·kg^−1^·min^−1^. A continuous infusion of vasopressin was initiated at 1.44 U/h, later titrated up to a maximum of 2.16 U/h. Heparin was reduced from 15.9 U/kg/min to 9.1 U/kg/min, representing an approximate 42% reduction, while activated clotting time (ACT) at 160 to 180 seconds and activated partial thromboplastin time (APTT) was maintained at 60 to 65 seconds.

**Figure 4.**
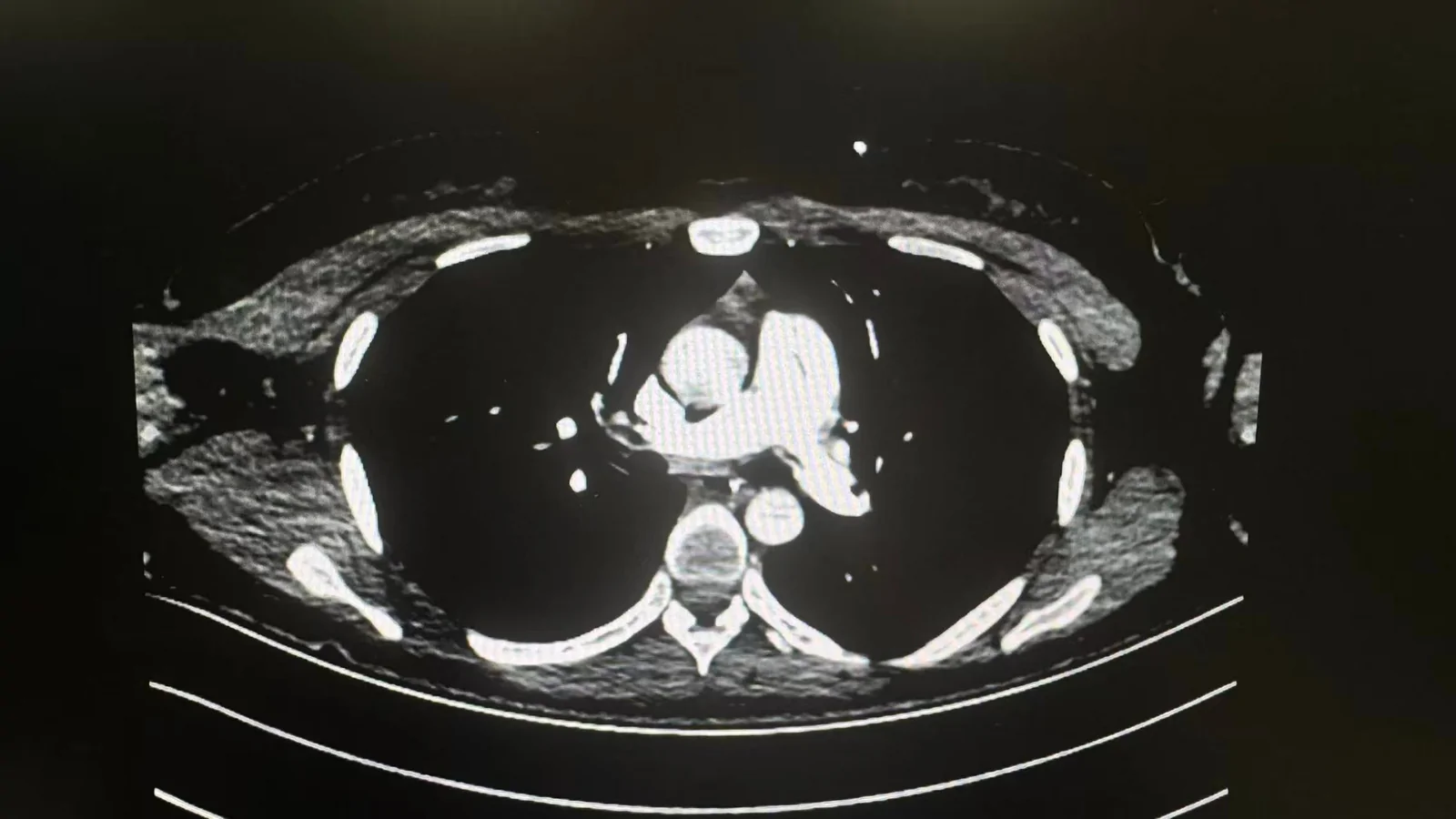
Follow-up CTPA after treatment showing complete resolution of pulmonary arterial thrombus. CTPA = computed tomography pulmonary angiography.

Airway management included suctioning as needed, with reduced negative pressure to minimize bronchial mucosal injury. Following these interventions, airway hemorrhage ceased completely within 6 hours, with a total blood loss of 120 mL. After 73 hours of VA-ECMO support, the patient was successfully weaned from ECMO. After 116 hours of mechanical ventilation, the patient regained consciousness and spontaneous respiration, allowing for successful extubation. Oxygen was administered via a nasal cannula at 5 L/min, with an pulse oxygen saturation of 97%. Repeat computed tomography pulmonary angiography demonstrated resolution of the thrombus at the main pulmonary artery bifurcation (Fig. 4). Follow-up transthoracic echocardiography revealed a right ventricular end-diastolic anteroposterior diameter (parasternal long-axis view) of 25 mm and a right ventricular basal end-diastolic diameter (A4C) of 32 mm. Electrocardiography no longer demonstrated the S_1_Q_3_T_3_ pattern, while D-dimer levels decreased to 2.074 mg/L.

### 2.3. Outcome and follow-up

Mild therapeutic hypothermia was maintained for neuroprotection throughout treatment. Bispectral index monitoring and serial assessment of neurological biomarkers were performed. Neuron-specific enolase levels ranged from 14.7 to 21.6 ng/mL, and S100β protein levels remained at <0.06 ng/mL. No complications involving the central nervous system or lower limb ischemia were observed. At the 1-month follow-up, transthoracic echocardiography demonstrated a right ventricular basal end-diastolic diameter (A4C) of 30 mm, pulmonary artery systolic pressure of 19 mm Hg, and left ventricular ejection fraction of 55%.

## 3. Discussion

Influenza A infection is an important risk factor for thrombosis, with an associated pulmonary embolism incidence of approximately 3.3%. The primary mechanisms include direct viral injury to the pulmonary vascular endothelium, activation of coagulation pathways due to an intense inflammatory response (“cytokine storm”), excessive platelet aggregation, and inhibition of fibrinolysis.^[4,5]^ Secondary contributing factors include dehydration leading to hemoconcentration and prolonged immobility resulting in venous stasis. These factors can culminate in a rapid, “cascade-like” increase in thrombus burden. Recently, our center treated 2 consecutive patients with acute high-risk pulmonary embolism following influenza A infection; this experience is consistent with prior findings that influenza infection is strongly associated with hypercoagulability and MPE.^[6]^ As such, this association warrants heightened clinical vigilance.

Reperfusion strategies for high-risk pulmonary embolism include systemic thrombolysis, surgical embolectomy, and catheter-directed interventions.^[7]^ ECMO is the preferred modality for mechanical circulatory support in patients with severe hemodynamic instability,^[8]^ as it provides a bridge to definitive reperfusion therapy. Other devices, such as right ventricular assist devices and Impella, are commonly limited by availability and are difficult to implement in primary or resource-limited hospitals. The incidence of severe bleeding during ECMO is approximately 30.9%, with airway hemorrhage accounting for 26% of all cases. Conservative management (cessation of anticoagulation combined with local measures alone) is associated with a mortality risk approaching 70%.^[8]^ The primary causes of airway hemorrhage during the perioperative period of ECMO include widespread viral-induced injury to the airway and pulmonary vascular endothelium, pulmonary infarction, procedural trauma from invasive interventions, anticoagulation-related coagulopathy during ECMO, and barotrauma from mechanical ventilation.^[9]^

Recent evidence from PubMed, as well as guidelines issued by the Extracorporeal Life Support Organization and the European Society of Intensive Care Medicine over the past 3 years, indicates that mainstream international management strategies remain predominantly conservative. Grading of airway hemorrhage is critical.^[10]^ For moderate-to-severe airway bleeding, the temporary cessation of anticoagulation is generally recommended; however, in mild cases, dose reduction of anticoagulation agents may be considered. Furthermore, protamine reversal may be used when necessary, while local hemostatic measures include bronchoscopic lavage, topical thrombin application, and antifibrinolytic therapy with tranexamic acid. However, these approaches raise significant concerns regarding increased thrombus burden, oxygenator thrombosis, recurrent pulmonary embolism, and even mortality.^[11,12]^ Some centers have attempted to discontinue mechanical ventilation with endotracheal tube clamping while maintaining full-flow ECMO support, thereby creating conditions for subsequent bronchial artery embolization or thrombus removal. This approach has been reported only in small cohorts at a limited number of centers, has not been widely adopted domestically, and still requires anticoagulation interruption, making it difficult to balance hemostasis and thrombosis prevention.

A review of the literature revealed no similar reports describing a strategy of continuous anticoagulation and thrombolysis combined with targeted hemostasis using vasopressin during airway hemorrhage in the perioperative period of ECMO. In the present case, anticoagulation was not completely discontinued. Instead, it was dynamically reduced and guided by ACT/APTT monitoring to balance bleeding control with ECMO circuit patency and thrombus prevention. Vasopressin was titrated as an adjunctive hemostatic strategy, and airway bleeding gradually ceased within 6 hours. This approach may provide a practical reference for selected ECMO patients in whom both hemorrhage and thrombotic risks are substantial. Dynamic monitoring of ACT and APTT, along with serial echocardiographic assessment of right ventricular function, demonstrated the gradual cessation of airway bleeding within 6 hours. This strategy of “continued anticoagulation and thrombolysis with targeted vasopressin-mediated hemostasis” avoided exacerbation of thrombus burden, reduced the risks of oxygenator exchange and infection, and lowered overall medical costs. Prior studies have shown that a VIS > 27 is associated with a 40.4% increase in adverse events, and that for every 10-point increase in VIS, mortality risk increases 1.0 to 1.2-fold, indicating that VIS may serve as a key indicator for prognosis assessment and therapeutic adjustment in such patients.^[13]^

Overall, in the present case of severe airway hemorrhage during the perioperative period of ECMO, heparin anticoagulation and urokinase thrombolysis were maintained without interruption, while the heparin dose was dynamically reduced and coagulation targets were adjusted according to the bleeding condition. A titrated vasopressin infusion was used as the core hemostatic strategy, combined with low-negative-pressure, as-needed airway suctioning, and close monitoring of coagulation parameters and VIS. This approach effectively controlled bleeding without any thrombotic complications, indicating that the treatment strategy employed in this case is both safe and feasible.

## 4. Conclusions

Although larger cohort studies are required to verify the study results, this case highlights that in patients with MPE requiring ECMO, a carefully balanced strategy of continued anticoagulation and thrombolysis combined with targeted vasopressin-mediated hemostasis may effectively control airway hemorrhage without increasing thrombotic risk.

## Author contributions

**Data curation:** Kun Cao.

**Investigation:** Kun Cao, Feng Hai.

**Writing – original draft:** Kun Cao.

**Writing – review & editing:** Feng Hai.
